# Effect of NAC and MPA ligands on growth process and optical properties of CdTe quantum dots

**DOI:** 10.1039/d5ra03961d

**Published:** 2025-09-10

**Authors:** Klara Verheles, Magdalena Wojtas, Artur Podhorodecki

**Affiliations:** a Department of Experimental Physics, Wroclaw University of Science and Technology Wybrzeze Wyspianskiego 27 50-370 Wroclaw Poland klara.verheles@pwr.edu.pl; b Department of Biochemistry, Wroclaw University of Science and Technology Wybrzeze Wyspianskiego 27 50-370 Wroclaw Poland

## Abstract

This study explores the aqueous synthesis of cadmium telluride (CdTe) quantum dots (QDs) using 3-mercaptopropionic acid (MPA) and *N*-acetyl-l-cysteine (NAC) as stabilizing ligands, focusing on their combined effect on QD growth, optical properties, and stability. By systematically varying the Cd : MPA ratio and MPA : NAC combinations, we identified an optimal Cd : MPA ratio of 1 : 1.75, yielding a quantum yield (QY) of ∼44.8%, and an MPA : NAC ratio of 1 : 2, achieving a superior QY of 66% with enhanced QD monodispersity. The dual-ligand system resulted in smaller particle sizes, slower growth kinetics, and a bathochromic shift in photoluminescence (from 567 nm to 731 nm) over 5–180 minutes of synthesis time. The synthesized QDs exhibited excellent photostability, with QY variations of less than 3% under 29 hours of continuous laser irradiation. Transmission electron microscopy (TEM) confirmed spherical, crystalline nanoparticles with diameters of 3.4–3.6 nm. These findings highlight the potential of MPA and NAC in tailoring CdTe QD properties, offering insights for their application in optoelectronics and biomedicine.

## Abstract 2

Dual-ligand stabilization of CdTe QDs using MPA and NAC enables precise size control and enhanced quantum yield (66%), achieving water-soluble, photostable nanocrystals with tunable NIR emission for biomedical and optoelectronic applications.

## Graphical abstract

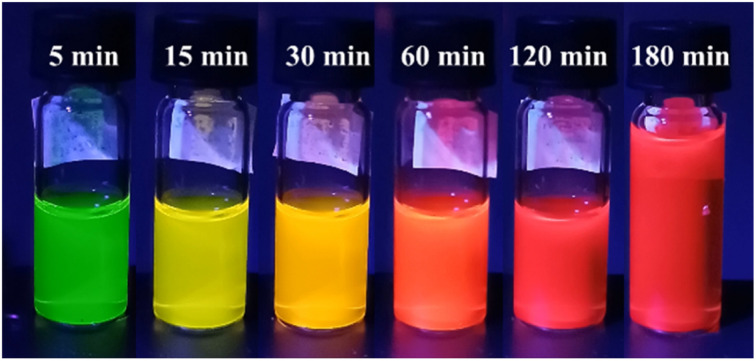

## Introduction

Cadmium telluride (CdTe) quantum dots (QDs) have attracted significant attention in recent years due to their unique size-dependent optical properties, high photoluminescence efficiency, and potential applications in fields such as bioimaging, sensing, and light-harvesting technologies.^1,2^ Compared to organic-phase synthesis, aqueous synthesis of CdTe QDs has become a preferred method due to its cost-effectiveness, environmental friendliness, and ability to produce water-dispersible nanoparticles suitable for biomedical applications.^3,4^ The stability and quantum yield (QY) of these QDs are critical parameters determining their practical applicability, and these properties are largely influenced by the choice of ligands used in the synthesis process.^5^

Among various investigated ligands, thiol-containing compounds such as 3-mercaptopropionic acid (MPA) and *N*-acetyl-l-cysteine (NAC) are widely employed as stabilizers in the aqueous synthesis of CdTe QDs.^6,7^ MPA, a short-chain thiol, is known for its ability to passivate the CdTe QD surface, enhancing their stability and fluorescence by reducing surface defects.^8^ Similarly, NAC, a biocompatible thiol with an additional acetyl functional group, improves the optical properties and colloidal stability of CdTe QDs, making them highly suitable for biomedical applications.^9–12^ Studies have shown that using MPA or NAC individually results in stable, luminescent CdTe QDs with moderate quantum yields. For example, CdTe QDs stabilized with MPA exhibit size-dependent fluorescence, making them promising for sensing and imaging applications, while NAC-capped CdTe QDs demonstrate reduced toxicity and improved biocompatibility.^13,14^ The influence of thiol ligand structure and Cd/thiol molar ratio on the optical characteristics of CdTe QDs has already been addressed in earlier studies,^15^ highlighting the importance of ligand chemistry in tuning their size and emission properties. However, these works focused on single-ligand systems and did not consider potential synergistic effects of mixed thiol stabilizers.

Despite extensive research on MPA and NAC as individual stabilizers, systematic studies on the combined use of these two thiols in CdTe QD synthesis are lacking in the literature. Currently, no published studies have explored how different combinations of MPA and NAC affect the synthesis, stability, and optical properties of CdTe QDs. On the other hand, synergistic effects between these ligands should lead to improved surface passivation and enhanced quantum yield compared to single-ligand systems. Recent advances in aqueous synthesis of CdTe QDs highlight the importance of ligand chemistry in tuning QD properties;^3,16,17^ however, the specific interactions between MPA and NAC in this context remain unexplored.

In this study, we investigate the effect of combining MPA and NAC on the aqueous synthesis and properties of CdTe QDs. Our findings demonstrate that this dual-ligand approach enables the formation of highly stable nanoparticles with significantly higher quantum yields than those synthesized with MPA or NAC alone, while also ensuring high optical stability of the resulting QDs. This effect is attributed to the complementary roles of both thiols in stabilizing the QD surface and optimizing their photophysical properties.^18^ By exploring this previously unstudied ligand combination, our research opens new possibilities for designing ligand systems to enhance CdTe QD efficiency, contributing to their broader application in nanotechnology and related fields.^19^

## Experimental

### Chemicals

Sodium borohydride (NaBH_4_, 99%), tellurium (30 mesh, 99.997%), cadmium chloride hemipentahydrate (CdCl_2_, 99.99%), 3-mercaptopropionic acid (MPA, 99%), *N*-acetyl-l-cysteine (NAC, 99%), sodium hydroxide (NaOH), distilled water.

### Synthetic procedures

#### Synthesis of CdTe quantum dots stabilized by MPA and NAC

##### Preparation of NaHTe precursor

The synthesis of NaHTe precursor was carried out in a three-neck flask under an inert atmosphere. Tellurium powder (51.04 mg) and NaBH_4_ (37.85 mg) were placed in the flask, and the system was subjected to vacuum for 10 minutes, followed by purging with argon for another 10 minutes. After that, 10 mL of distilled water was added under continuous stirring at 600 rpm. The system was again evacuated for 10 minutes and subsequently purged with argon for 10 minutes. The reaction mixture was then heated to 80 °C while stirring, and once the temperature was reached, the stirring continued for an additional 30 minutes. The resulting NaHTe solution exhibited a dark burgundy-red color.^4^

##### Preparation of Cd-ligand complex

In a separate three-neck flask, CdCl_2_ (36.6 mg) was mixed with varying volumes of 3-mercaptopropionic acid (MPA), specifically 17.4, 26.1, 30.45, and 34.8 μL, corresponding to Cd : MPA molar ratios of 1 : 1, 1 : 1.5, 1 : 1.75, and 1 : 2, respectively. The mixture was then diluted with 40 mL of distilled water and stirred at 600 rpm. The pH of the solution was adjusted to 12 using 1 M NaOH solution.^5^ The system was subsequently subjected to vacuum for 10 minutes and purged with argon for another 10 minutes.

##### Synthesis of CdTe quantum dots

Under an argon atmosphere, 0.19 mL of freshly prepared NaHTe solution was injected into the Cd-ligand solution, leading to an immediate color change from transparent to golden yellow. The system was further degassed under vacuum for 10 minutes and then purged with argon for another 10 minutes. The reaction mixture was heated to 100 °C while stirring, and the reaction time was counted from the moment the temperature reached 100 °C. Aliquots were collected at 5, 15, 30, 60, 120, and 180 minutes to monitor the growth of quantum dots.^6^

##### Purification of CdTe quantum dots

The synthesized CdTe quantum dots were purified by adding twice the volume of isopropanol to the reaction solution, followed by vigorous mixing. The suspension was centrifuged at 6000 rpm for 20 minutes. The supernatant was discarded, and the precipitate was redispersed in distilled water. This purification step was performed once. The samples were stored at room temperature for further characterization.

##### Optimization and ligand variation

Based on the preliminary results, the Cd : MPA ratio of 1 : 1.75 was found to be optimal for achieving high quantum yield and stability. Subsequently, an additional set of experiments was conducted where the total ligand concentration was maintained at the Cd : ligand ratio of 1 : 1.75, but different combinations of MPA and NAC were used: 1 : 0, 2 : 1, 1 : 2, and 0 : 1. The synthesis procedure remained the same as described above. Notably, when Te precursor was added to the CdCl_2_ solution containing both MPA and NAC, the color of the reaction mixture turned a darker golden-orange compared to the golden-yellow observed in the presence of MPA alone.

## Results and discussion

### The effect of Cd : MPA ratio on the optical properties of CdTe quantum dots

**Fig. 1:**
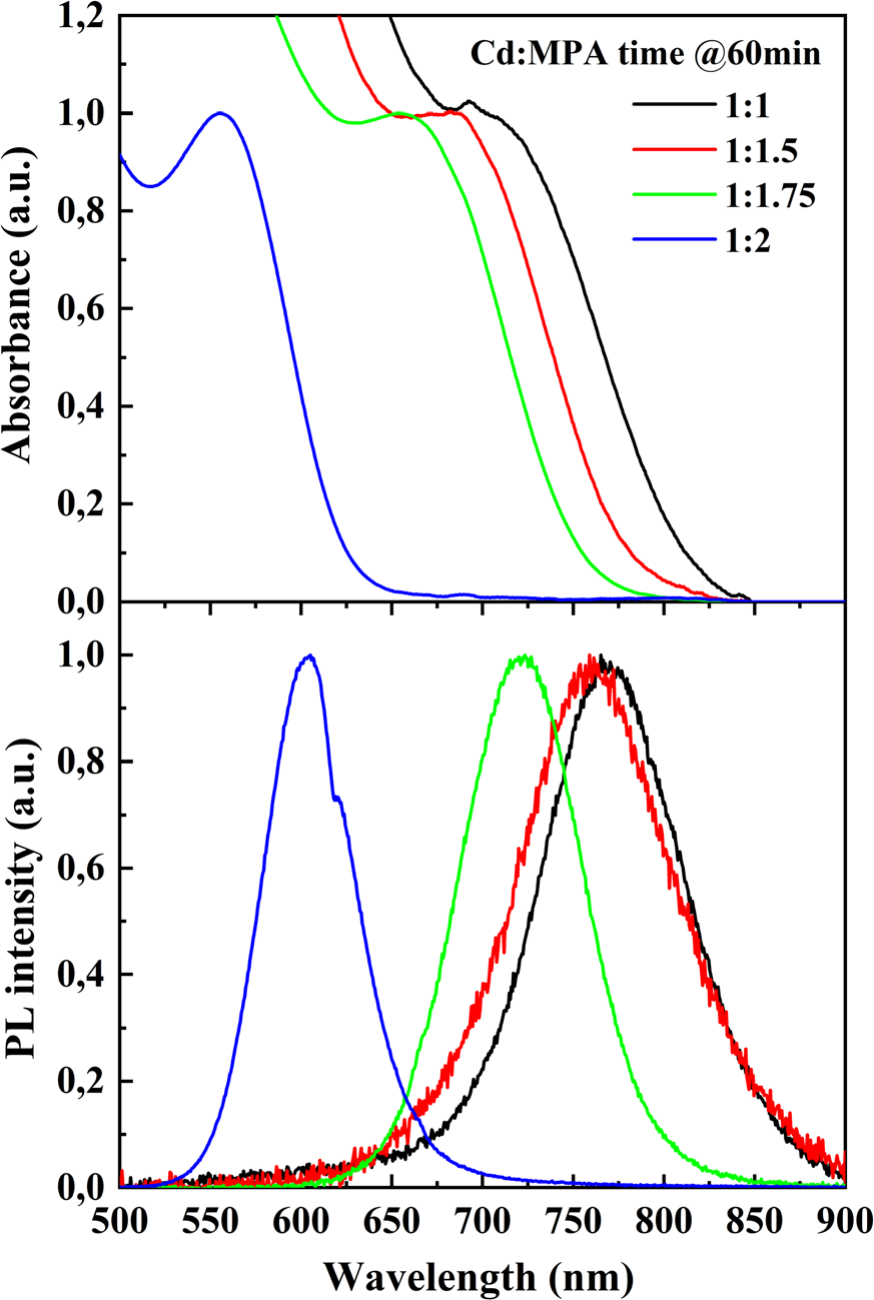
Absorption spectra (top) and normalized photoluminescence spectra (bottom) of CdTe quantum dots synthesized at different Cd : MPA ratios (1 : 1, 1 : 1.5, 1 : 1.75 and 1 : 2) after 60 minutes of heating at 100 °C.

Fig. 1 presents normalized absorption and photoluminescence spectra of CdTe quantum dots synthesized at different Cd : MPA ratios (1 : 1; 1 : 1.5; 1 : 1.75; 1 : 2) and collected 60 minutes after heating at 100 °C.

From Fig. 1 it can be seen that as the amount of MPA increases, the absorption peak shifts toward shorter wavelengths (blue shift), indicating the formation of smaller particles.^20^ The maxima of photoluminescence spectra exhibit a similar shift, transitioning from 768 nm at Cd : MPA = 1 : 1 to 603 nm at Cd : MPA = 1 : 2.

**Fig. 2:**
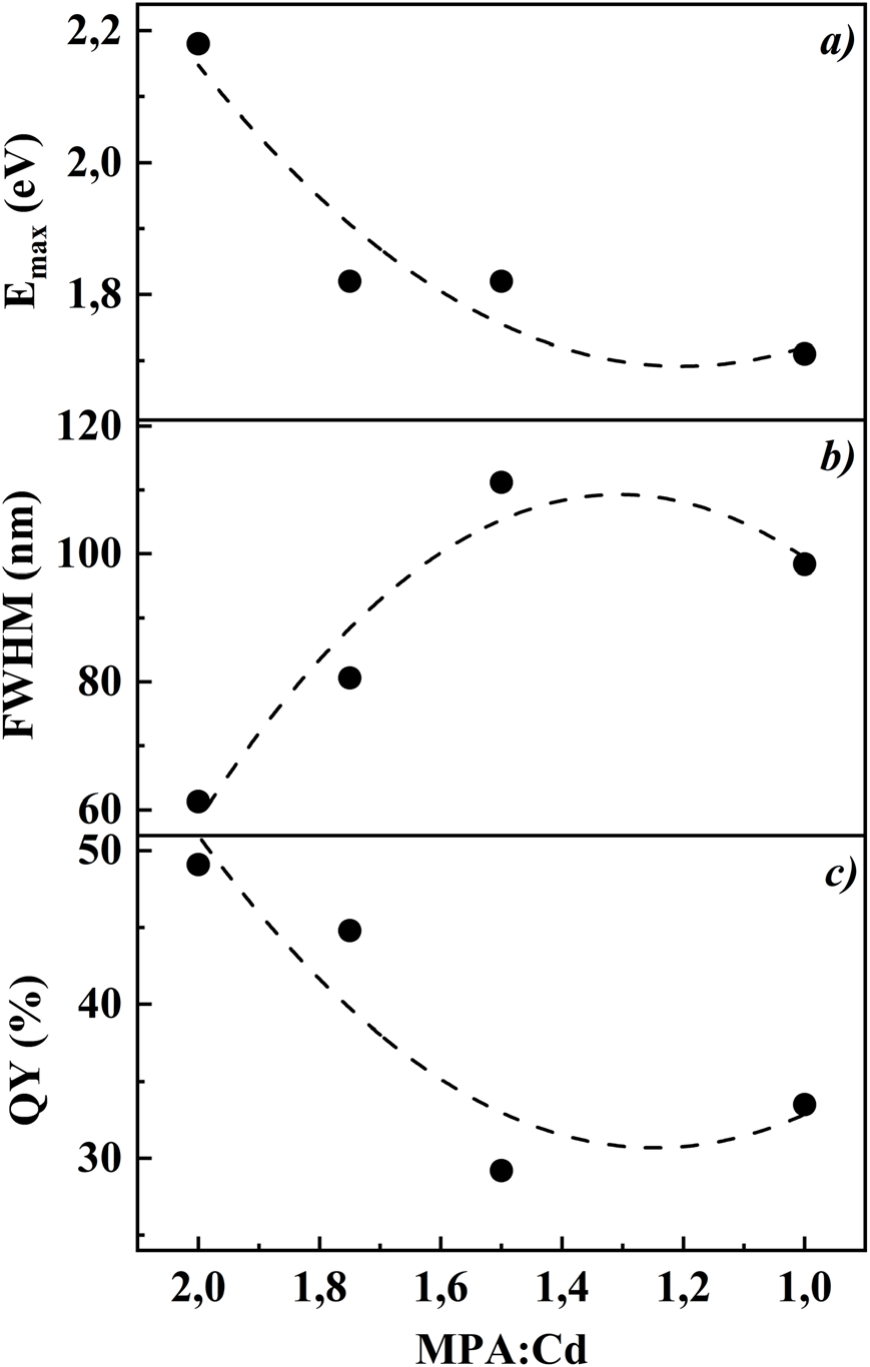
(a) Dependence of peak absorption energy, (b) full width at half maximum (FWHM) of the photoluminescence spectra, and (c) quantum yield of CdTe quantum dots on the amount of MPA at different Cd : MPA ratios (1 : 2, 1 : 1.75, 1 : 1.5 and 1 : 1) after 60 minutes of heating at 100 °C.

Fig. 2 presents the dependence of CdTe QDs absorption peak position, full width at half maximum (FWHM) of the photoluminescence spectra, and quantum yield as a function of MPA concentration. The absorption energy increases with higher MPA content, confirming the reduction in particle size.^21^ The minimum FWHM of the photoluminescence spectra is observed at Cd : MPA = 1 : 1.75 (∼80 nm), indicating the highest QD monodispersity,^22^ and also at this ratio the quantum yield reaches its maximum value ∼44.8%. These results suggest that the CdTe quantum dots synthesized at Cd : MPA = 1 : 1.75 exhibit the best optical quality.

### Influence of MPA and NAC compound on the properties of CdTe quantum dots

**Fig. 3:**
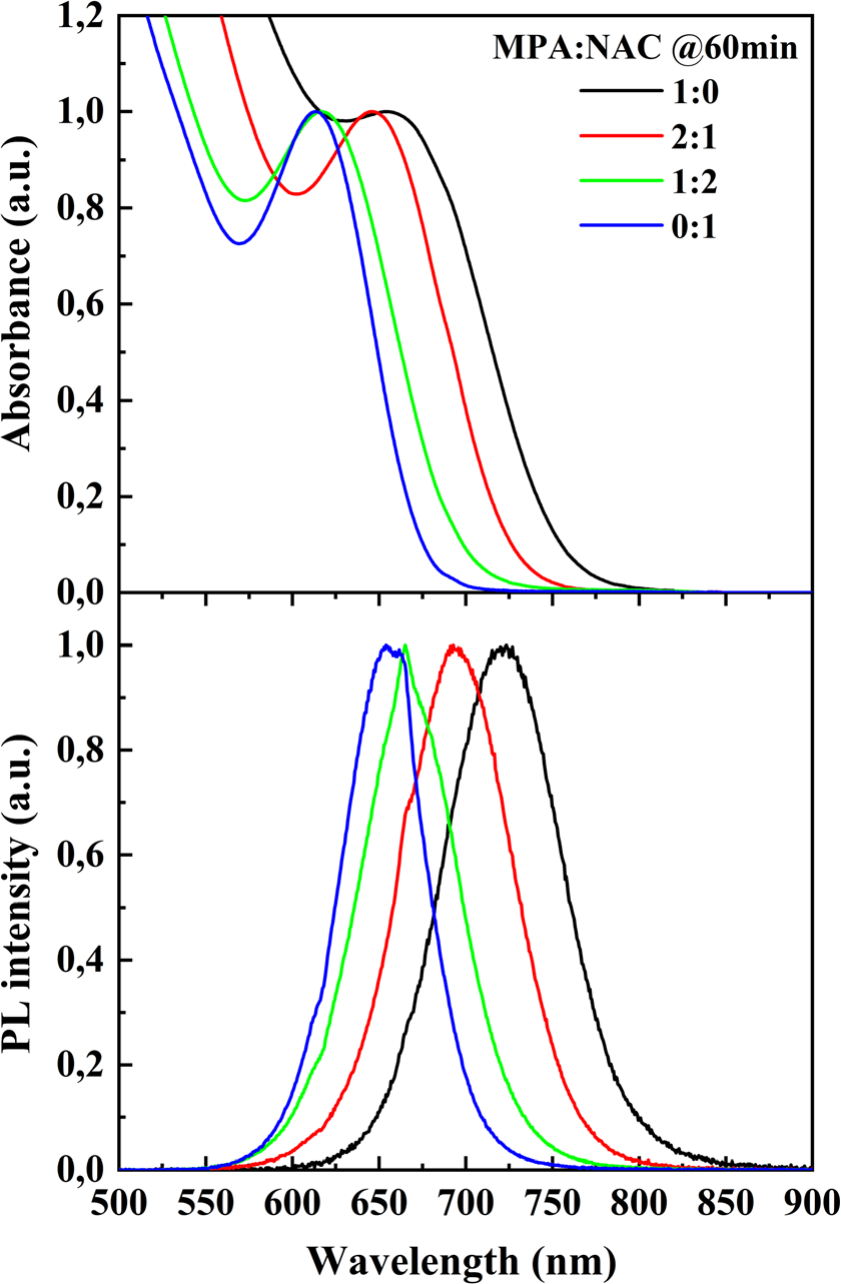
Absorption spectra (top) and normalized photoluminescence spectra (bottom) of CdTe quantum dots synthesized at a Cd : acid ratio of 1 : 1.75 with different MPA : NAC ratios (1 : 0, 2 : 1, 1 : 2, 0 : 1) after 60 minutes of heating at 100 °C.

Since the Cd : MPA ratio of 1 : 1.75 demonstrated the best results, further synthesis was carried out with the addition of *N*-acetyl-l-cysteine (NAC) while maintaining a fixed total amount of acids. The absorption and normalized photoluminescence spectra are presented in Fig. 3.

The addition of NAC leads to a blue shift in both the absorption and photoluminescence spectra, indicating the formation of smaller-sized particles.^23^ The photoluminescence maxima for samples with MPA : NAC ratios of 1 : 0, 2 : 1, 1 : 2, and 0 : 1, collected after 60 minutes of heating at 100 °C, are 723, 684, 665, and 656 nm, respectively.

**Fig. 4:**
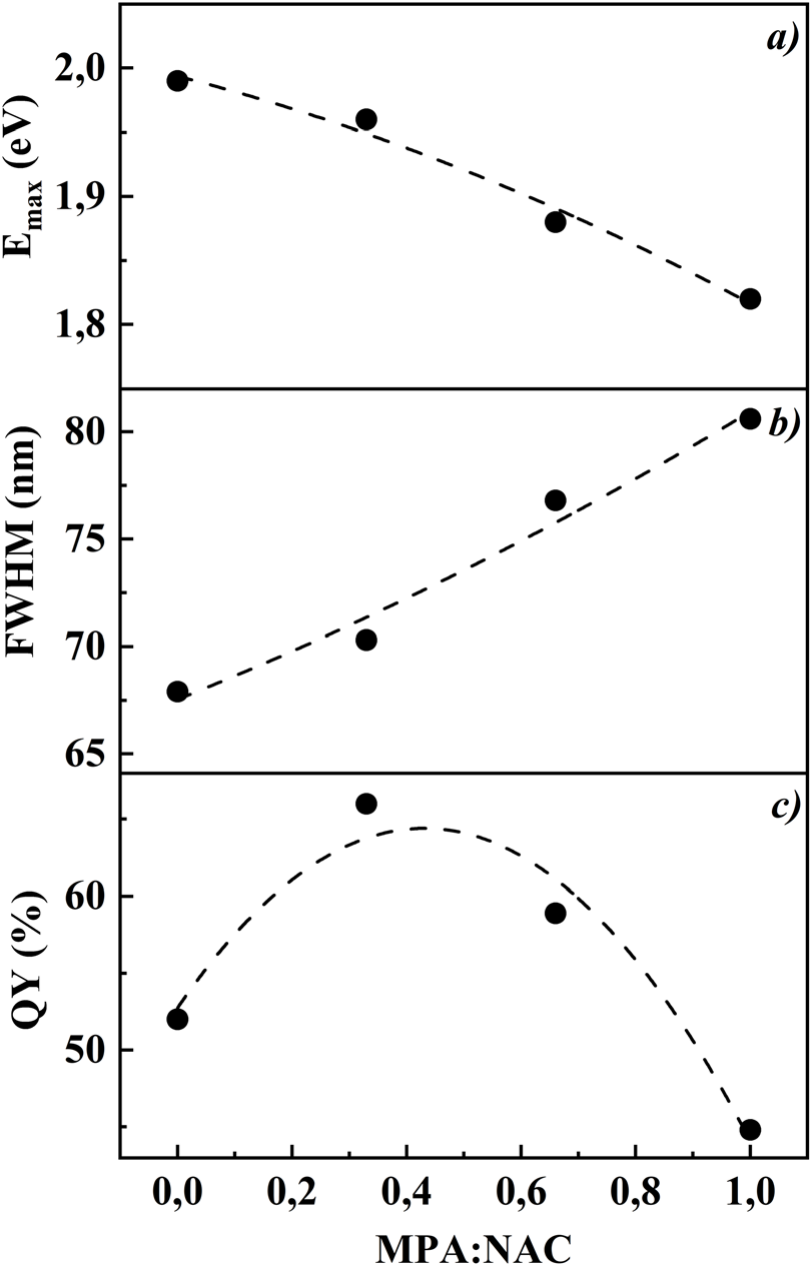
(a) Dependence of peak absorption energy, (b) full width at half maximum (FWHM) of photoluminescence spectra, and (c) quantum yield of CdTe quantum dots on the MPA : NAC ratio (0 : 1, 1 : 2, 2 : 1, 1 : 0) after 60 minutes of heating at 100 °C.

Fig. 4 presents the dependencies of peak absorption energy, FWHM, and quantum yield on the MPA : NAC ratio. The peak absorption energy increases with a higher NAC part, confirming the reduction in particle size. The low FWHM is observed at MPA : NAC = 1 : 2 (70.3 nm), indicating the high monodispersity. The maximum quantum yield is also achieved at MPA : NAC = 1 : 2 (66%), which is significantly higher than when using only MPA (∼44.8%).

The observed differences in optical properties of the CdTe quantum dots can be attributed to the chemical structure and binding behavior of the capping ligands used—3-mercaptopropionic acid and *N*-acetyl-l-cysteine. MPA, a small thiol-containing molecule, facilitates rapid nucleation and relatively uniform growth due to strong coordination with Cd^2+^ ions and minimal steric hindrance.^4,18,24^ As the Cd : MPA ratio increases (from 1 : 1 to 1 : 2), the absorption and photoluminescence maxima exhibit a blue shift, which corresponds to the formation of smaller-sized QDs. In contrast, NAC contains additional functional groups, including an amide and a carboxyl group, which can engage in hydrogen bonding and alter the local environment of the particle surface.^25,26^ These structural features may lead to slower nucleation and more controlled growth, resulting in smaller and more uniform particles at certain ratios.

The combination of MPA and NAC provides synergistic effects: MPA ensures effective passivation during the early stages of growth, while NAC enhances long-term colloidal and optical stability due to its complex binding capabilities. The blue shift observed in the absorption and PL spectra for mixed-ligand systems (particularly MPA : NAC = 1 : 2) suggests that NAC limits particle growth more effectively than MPA alone, which is also reflected in the narrower photoluminescence spectra (lower FWHM) and increased quantum yield (up to 66%).

Compared to earlier studies that focused on single-ligand systems,^27^ our results demonstrate that a binary ligand approach allows for more precise control over nanocrystal size and surface passivation, resulting in narrower PL spectra and enhanced quantum yield.

These findings indicate that both the steric and electronic properties of the ligands play a key role in determining the final size, monodispersity, and emission characteristics of the CdTe QDs.

### Effect of synthesis time on quantum dot properties at MPA : NAC = 1 : 2

**Fig. 5:**
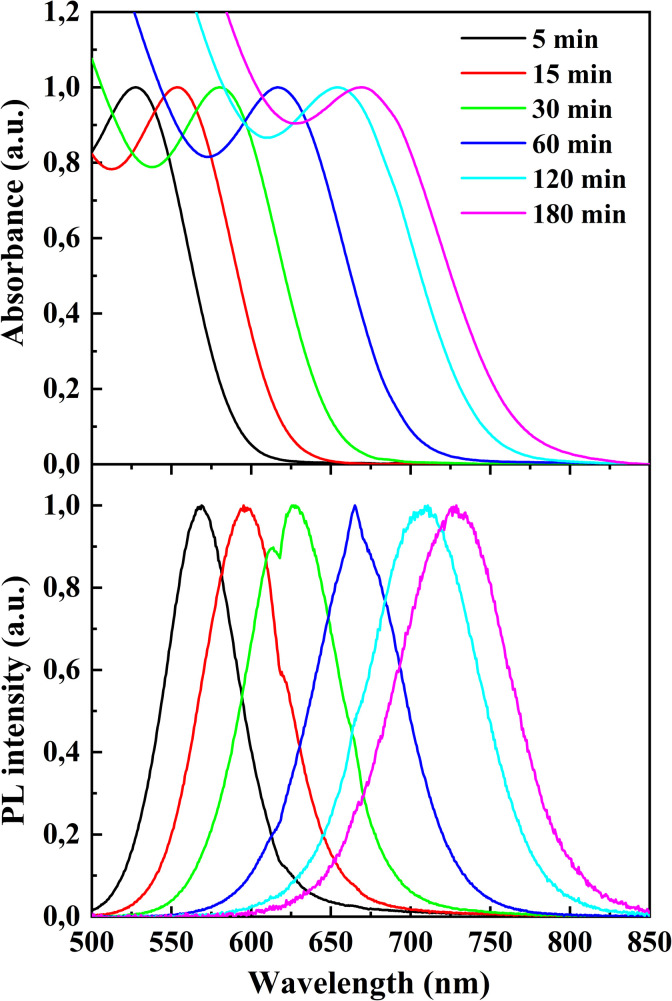
Absorption spectra (top) and normalized photoluminescence spectra (bottom) of CdTe quantum dots synthesized at an MPA : NAC ratio of 1 : 2, collected at different times after heating at 100 °C (5, 15, 30, 60, 120, and 180 minutes).

Fig. 5 presents the absorption spectra and photoluminescence spectra of CdTe quantum dots sampled at 5, 15, 30, 60, 120, and 180 minutes after the start of heating at 100 °C.

The photoluminescence peak shifts from 567 nm (5 minutes) to 731 nm (180 minutes), while the photoluminescence intensity initially increases (peaking at 15–60 minutes) and then begins to decline, which may be associated with particle growth.

**Fig. 6:**
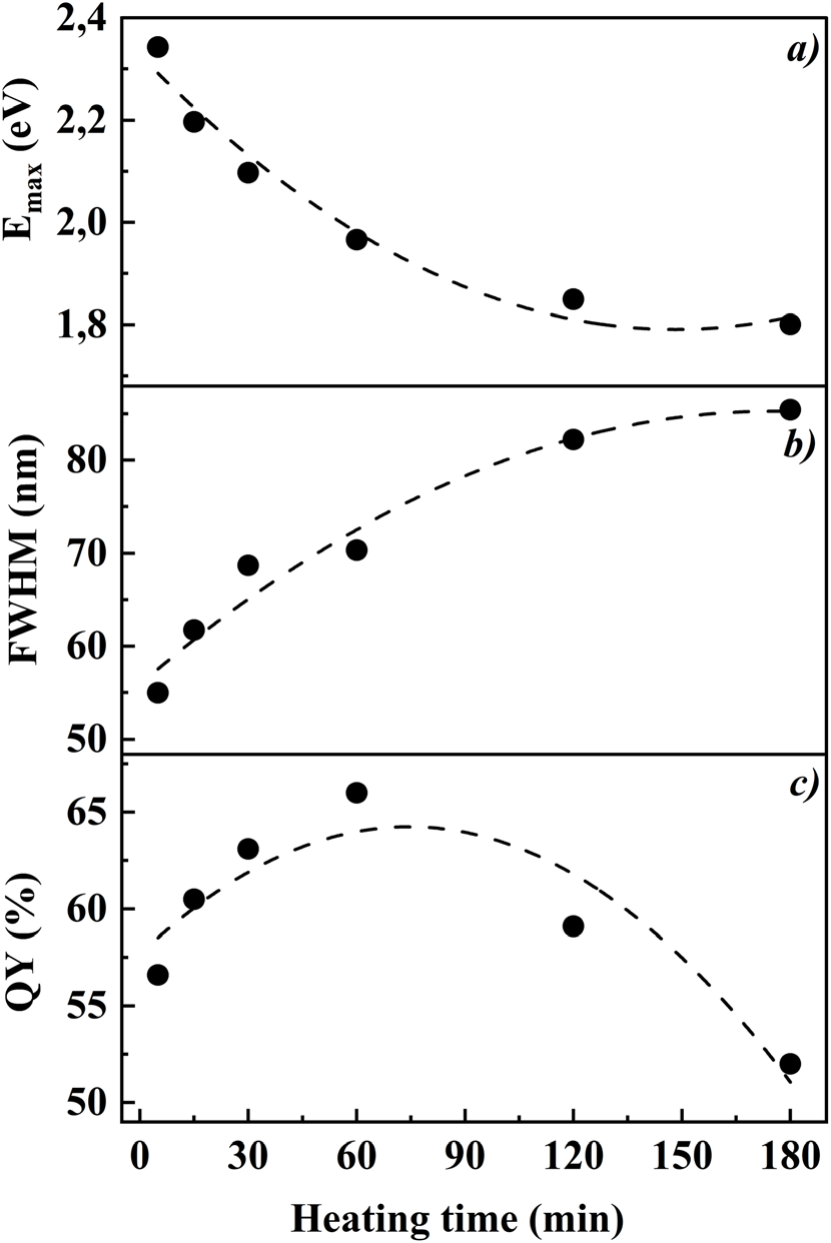
(a) Dependence of peak absorption energy, (b) full width at half maximum (FWHM) of the photoluminescence spectra, and (c) quantum yield of CdTe quantum dots synthesized at an MPA : NAC ratio of 1 : 2 on heating time at 100 °C (5, 15, 30, 60, 120, and 180 minutes).

Fig. 6 presents the dependencies of peak absorption energy, FWHM, and quantum yield on synthesis time. The peak absorption energy decreases with increasing synthesis time, confirming particle growth. The FWHM of the photoluminescence spectra increases from ∼55 nm (5 minutes) to 85 nm (180 minutes), indicating an increase in particle polydispersity. The quantum yield reaches a maximum (66%) at 60 minutes and then begins to decline.

**Fig. 7:**
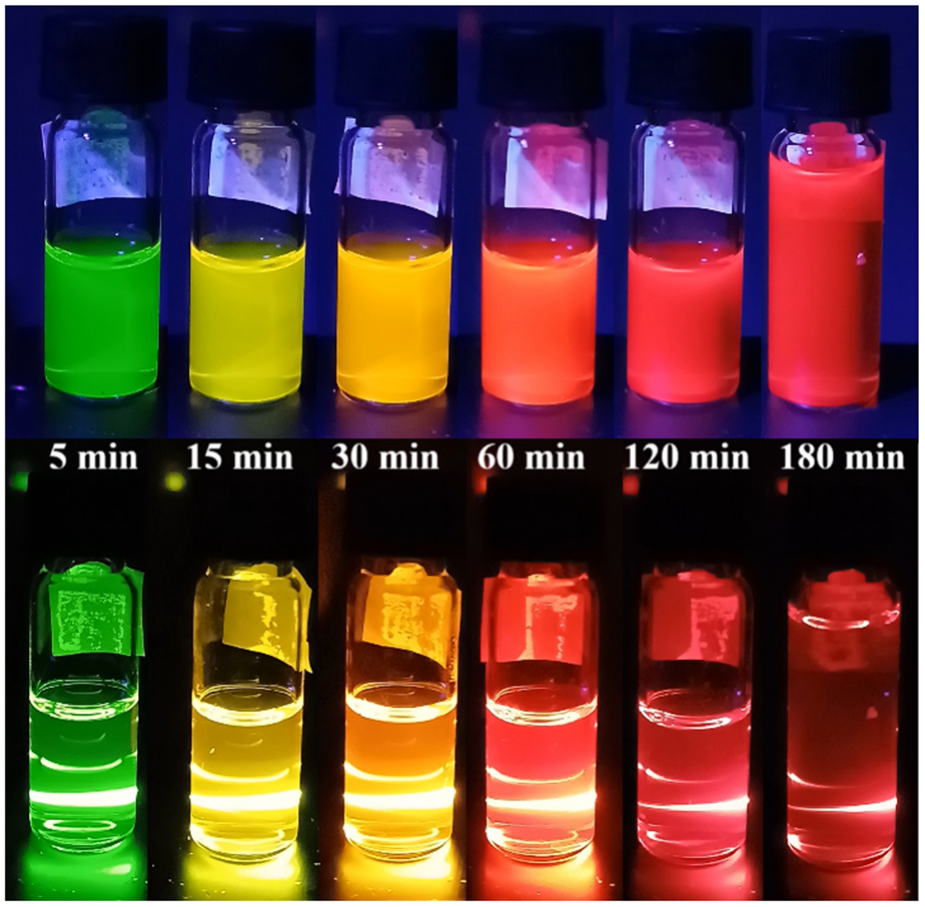
Photographs of CdTe quantum dots synthesized at an MPA : NAC ratio of 1 : 2 at different reaction times (5, 15, 30, 60, 120, and 180 minutes) under UV lamp illumination (top) and 405 nm laser illumination (bottom).

Fig. 7 presents photographs of CdTe quantum dot solutions synthesized at an MPA : NAC ratio of 1 : 2 at different time points (5, 15, 30, 60, 120, and 180 minutes). The samples were illuminated with a UV lamp (left) and a 405 nm laser (right). It is evident that as the reaction time increases, the luminescence color shifts from green (5 min) to red (180 min). At later synthesis stages (120 and 180 min), the emission becomes less visible, which is associated with the transition of luminescence into the infrared region, where human eye sensitivity is significantly lower.

Several previous studies have reported the optical properties of CdTe QDs capped individually with MPA or NAC. For instance, NAC-capped QDs prepared *via* hydrothermal methods showed QY up to 45%, depending on synthesis conditions.^28,29^ CdTe QDs capped solely with MPA typically emit around 570–690 nm with a QY of ∼50%.^4^ In comparison, our dual-ligand system (MPA : NAC = 1 : 2) demonstrated superior performance, yielding stable QDs with a QY of 66% and an emission of 665 nm under mild aqueous conditions and without the use of high-temperature hydrothermal synthesis. This highlights the efficiency of combining MPA and NAC in improving surface passivation and optical output of CdTe nanocrystals.

### Photostability of CdTe quantum dots

The emission quantum yield is one of the most important parameters when characterizing optically active materials. However, from a practical point of view, the optical stability of colloidal QDs is even more critical. It is well known that this type of nanomaterial can degrade over time due to various factors, and even QDs with a quantum yield close to unity can become practically useless if this value drops under light exposure with time. Therefore, to assess the practical potential of the synthesized QDs, we also evaluated their optical stability—an aspect that, for some reason, is rarely reported in other publications.

**Fig. 8:**
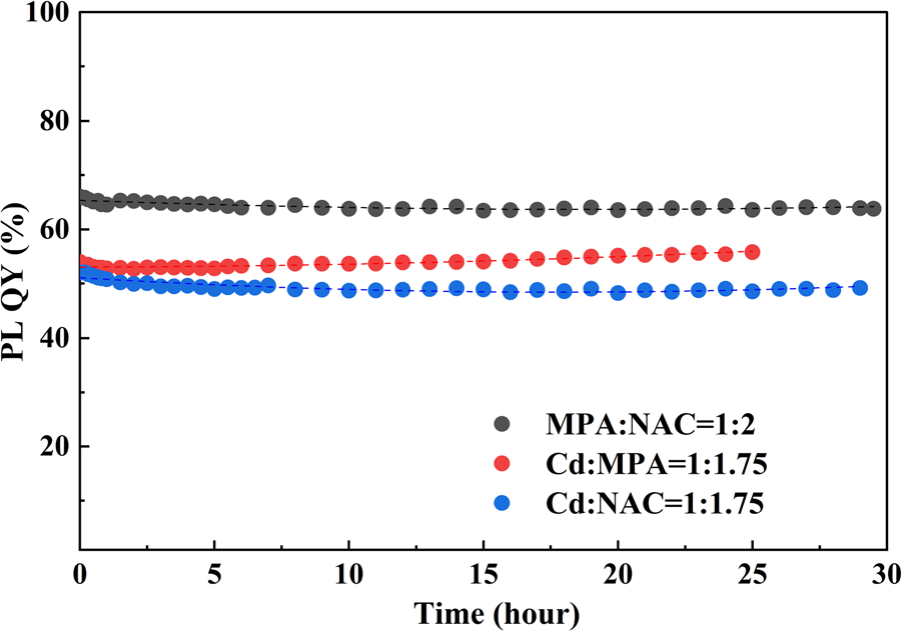
Dependence of the quantum yield of CdTe quantum dots (with different ligands: Cd : MPA = 1 : 1.75 (*λ* = 653 nm); MPA : NAC = 1 : 2 (*λ* = 665 nm); Cd : NAC = 1 : 1.75 (*λ* = 660 nm)) on time under continuous laser irradiation for 29 hours. The samples were irradiated using a 405 nm laser with a power of 200 mW.

To evaluate the stability of the quantum yield, nanoparticles with different ligand compositions (Cd : MPA = 1 : 1.75; MPA : NAC = 1 : 2; Cd : NAC = 1 : 1.75) were exposed to laser irradiation for 25–29 hours. The change in quantum yield over time is presented in Fig. 8.

All samples exhibit high photostability: the quantum yield varies by no more than 1–3% throughout the experiment. This confirms that the combination of MPA and NAC promotes the formation of stable CdTe quantum dots with high resistance to photodegradation. Moreover, the obtained QDs also possess very high colloidal stability, and no agglomeration has been observed over a period of more than 3 months.

### TEM images of CdTe quantum dots

**Fig. 9:**
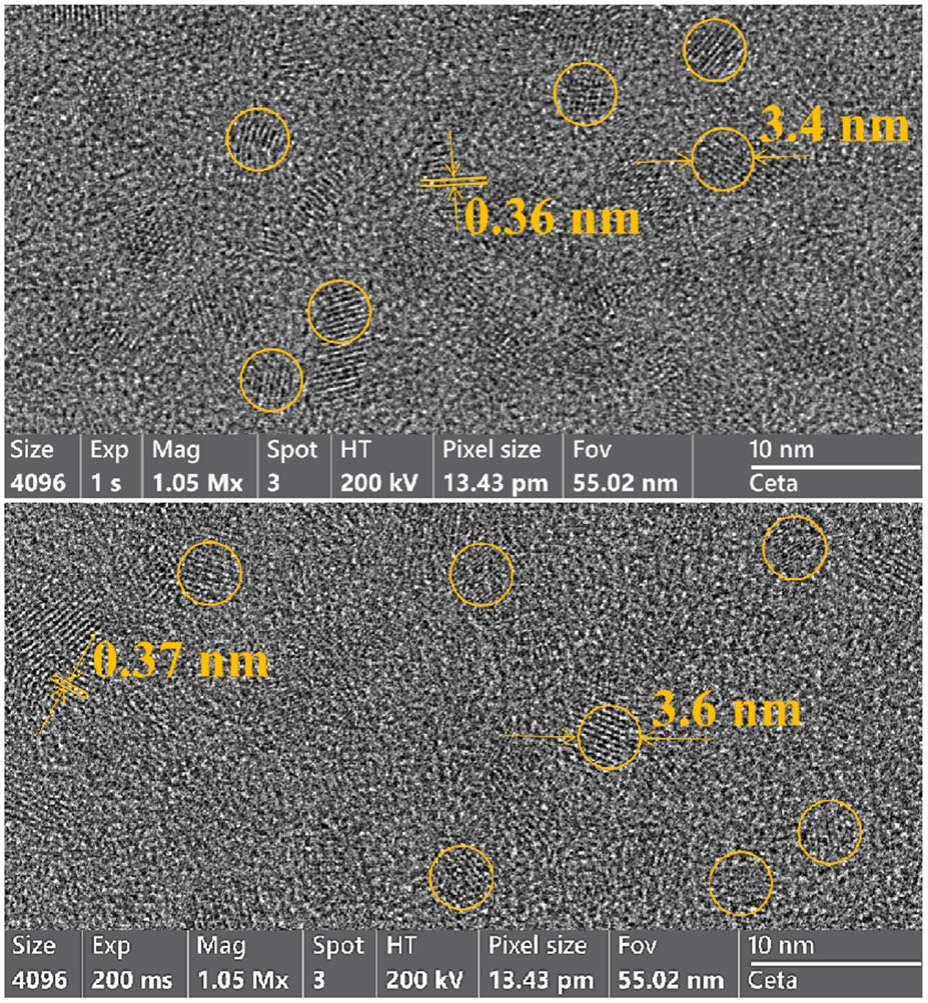
TEM images of CdTe quantum dots synthesized at Cd : MPA = 1 : 1.75 (*λ* = 653 nm) after 15 minutes of heating at 100 °C (top) and MPA : NAC = 1 : 2 (*λ* = 665 nm) after 60 minutes of heating at 100 °C (bottom).

Fig. 9 presents TEM images of CdTe quantum dots synthesized under different conditions. In both cases, the particles exhibit a spherical shape and demonstrate good crystallinity.

For the sample synthesized at a Cd : MPA ratio of 1 : 1.75 (*λ* = 653 nm, 15 minutes after reaching 100 °C), the average particle diameter is 3.4 nm, and the *d*-spacing value of 0.36 nm for (111) plane.^11^

For the sample obtained at an MPA : NAC ratio of 1 : 2 (*λ* = 665 nm, 60 minutes after reaching 100 °C), the particle diameter increases to 3.6 nm, and the *d*-spacing expands to 0.37 nm.

The TEM results confirm that the synthesized CdTe quantum dots possess nanometer-scale dimensions and a high degree of structural order. The slight increase in particle diameter when substituting part of MPA with NAC correlates with the observed red shift in the photoluminescence spectra. The measured *d*-spacing values align with the crystalline structure of CdTe, confirming the formation of well-defined nanoparticles.

### Zeta potential analysis

The colloidal stability of CdTe QDs capped with different ligands was evaluated by measuring the zeta potential at 25 °C in water. Each sample was measured in triplicate, and the average value was recorded. The results revealed that all samples exhibited high negative zeta potentials, indicating strong electrostatic repulsion and stable dispersion in aqueous media. Specifically, QDs synthesized with MPA : NAC (1 : 2) exhibited the highest zeta potential value of −43.5 mV, compared to −32.7 mV for MPA alone and −36.8 mV for NAC. These findings confirm the enhanced stabilizing effect of the dual-ligand system, contributing to improved colloidal stability of the nanocrystals in water.

### X-ray diffraction analysis

X-ray diffraction (XRD) analysis of CdTe nanoparticles, synthesized with a ligand combination of MPA : NAC at a 1 : 2 ratio, was performed using a Malvern Panalytical Empyrean diffractometer equipped with a CuKα_1_ X-ray source (*λ* = 1.540 598 Å). The aqueous nanoparticle suspension was loaded into a quartz capillary and mounted on a spinning stage for uniform exposure. Diffraction data were collected in the *θ*–2*θ* configuration over a 2*θ* range of 10° to 110° using a Pixcel3D detector. Beam collimation was optimized with a linear X-ray focus and an anti-scatter slit.

**Fig. 10:**
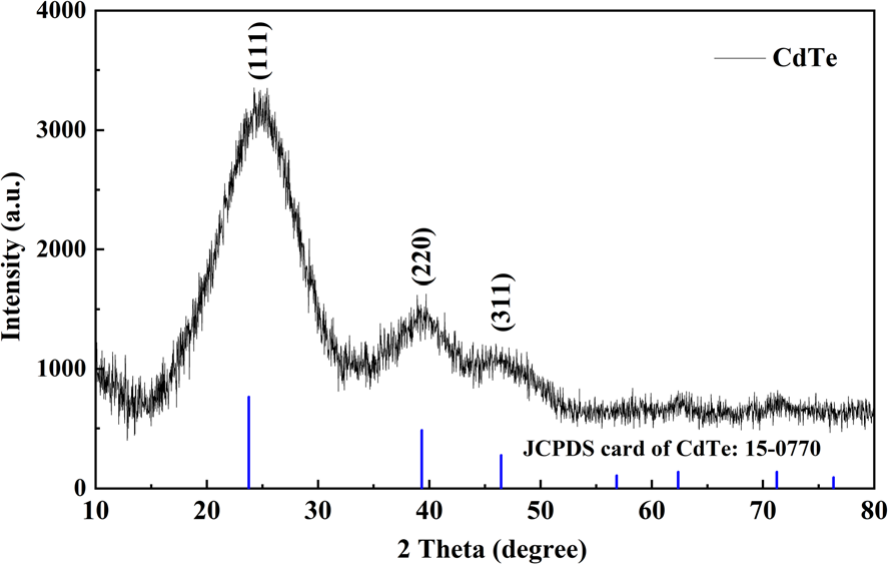
XRD patterns of CdTe quantum dots synthesized at MPA : NAC = 1 : 2 (*λ* = 665 nm) after 60 minutes of heating at 100 °C. The line XRD spectra correspond to bulk cubic zinc blende CdTe (blue lines at the bottom).

The obtained diffraction pattern (Fig. 10) displays three characteristic reflections at 2*θ* = 24.2°, 39.3°, and 46.4°. The most intense peak at 24.2° corresponds to the (111) lattice plane, while the weaker peaks at 39.3° and 46.4° are assigned to the (220) and (311) planes, respectively. For comparison, the reference peak positions from JCPDS card No. 15-0770 (shown in blue) are also plotted, corresponding to the cubic (zinc blende) CdTe structure.

The good agreement between the experimental data and the reference values confirms that the synthesized QDs predominantly crystallize in the cubic modification of CdTe. The broadening of the diffraction peaks relative to bulk crystals is characteristic of nanomaterials and indicates the presence of small crystallites. This observation is consistent with the TEM analysis (Fig. 9), which revealed particle sizes of 3.4–3.6 nm, in line with the expected nanoscale nature of the synthesized QDs.

Overall, the XRD results validate the successful formation of nanocrystalline CdTe with a cubic crystal structure, complementing the optical data (Fig. 1–4) and colloidal stability measurements (ζ-potential, −43.5 mV), and demonstrating the effectiveness of the MPA : NAC = 1 : 2 ligand system in producing stable, high-quality nanocrystals.

## Conclusions

In this work, the aqueous synthesis of CdTe quantum dots using various ratios of 3-mercaptopropionic acid (MPA) and *N*-acetyl-l-cysteine (NAC) was carried out, allowing the study of the influence of these ligands on their optical properties. Optimization of the Cd : MPA ratio showed that the most narrow size distribution of particles and the highest quantum yield (44.8%) were achieved at Cd : MPA = 1 : 1.75.

The use of the MPA and NAC combination significantly improved the characteristics of the quantum dots. The highest quantum yield values (66%) and narrow size distribution were observed at MPA : NAC = 1 : 2. As the particle growth time increased from 5 to 180 minutes, a bathochromic shift in luminescence was observed (from 567 nm to 731 nm), confirming particle growth, and the quantum yield reached its maximum at 60 minutes.

CdTe quantum dots synthesized with the MPA and NAC combination demonstrated high luminescence stability, maintaining a quantum yield within 1–3% during prolonged laser irradiation (405 nm) for 29 hours. Visual observations under UV light and a laser confirmed the systematic change in luminescence color, and TEM images showed that particles synthesized under optimal conditions were spherical in shape and highly uniform.

Thus, it was found that the combination of MPA and NAC in the aqueous synthesis of CdTe quantum dots leads to nanoparticles with improved quantum efficiency, narrow size distribution, and high photostability, making them promising for optoelectronic and biomedical applications.

## Author contributions

Klara Verheles: conceptualization, methodology, investigation, data curation, formal analysis, visualization, writing – original draft. Artur Podhorodecki: supervision, project administration, writing – review and editing, funding acquisition. Magdalena Wojtas: resources (TEM imaging).

## Conflicts of interest

There are no conflicts to declare.

## Data Availability

All data underlying the results are available as part of the article and no additional source data are required.
